# Management of Venous Thromboembolism (VTE) Complication Following Limb Replantation: A Report of Two Cases and Literature Review

**DOI:** 10.7759/cureus.94321

**Published:** 2025-10-10

**Authors:** Jingjing Wen, Zhegang Zhou, Johnson Boey, Fanbin Meng, Longbiao Yu

**Affiliations:** 1 Department of Microsurgery and Lymphatic Surgery, Peking University Shenzhen Hospital, Shenzhen, CHN; 2 Department of Podiatry, SingHealth Polyclinics, Singapore, SGP; 3 Department of Hand, Foot, and Vascular Surgery, Peking University Shenzhen Hospital, Shenzhen, CHN

**Keywords:** anticoagulation, deep vein thrombosis (dvt), limb replantation, pulmonary embolism (pe), venous filters

## Abstract

Venous thromboembolism (VTE) frequently occurs as a perioperative complication following major soft tissue reconstruction. Deep vein thrombosis (DVT) and pulmonary embolism (PE) represent the prevalent forms of VTE. Most of the symptoms manifest in a subtle or asymptomatic manner, complicating accurate and timely diagnosis. Upon identification, patients require prompt and careful management as a delay in diagnosis has been associated with high mortality and morbidity. The management of VTE involves balancing adequate anticoagulation and the associated risk of bleeding.

This paper presents two cases of open fracture accompanied by vascular injuries resulting from high-energy trauma. Surgical debridement, vascular repair, and limb replantation were performed promptly. Negative pressure wound therapy (NPWT) was adopted to optimize the wound bed before coverage with a perforator-free flap. To mitigate the risk of bleeding associated with NPWT, anticoagulation was implemented using dextran. PE and concomitant DVT were identified within three days following extensive reconstructive procedures. Low-molecular-weight heparin replaced dextran, resulting in the resolution of VTE events within one week.

The clinical team's prompt response has averted more serious perioperative complications. It is essential for the surgical team to conduct thorough surgical planning, which includes assessing the risk of VTE, determining the duration and type of the surgery involved, and anticipating potential perioperative complications.

## Introduction

Venous thromboembolism (VTE) is a prevalent vascular complication characterized by deep vein thrombosis (DVT) and pulmonary embolism (PE), both of which share a common pathophysiological mechanism. PE generally arises from DVT, where an abnormal thrombus dislodges from the endothelial lining and traverses the bloodstream as an embolus, ultimately occluding small-caliber pulmonary arteries in the lungs. The annual incidence of VTE ranges from 1 to 2 cases per 1,000 individuals per year, with DVT occurring at double the rate of PEs [1]. However, the manifestations of VTE are frequently underestimated or asymptomatic, particularly in instances of DVT [2]. Initial clinical signs are typically associated with PE. In some cases, DVT occurs concurrently with PE, and this association is correlated with a more severe presentation of PE [2,3]. The implication of this coexistence complicates diagnosis.

The aetiology of VTE is complex, involving a combination of demographic, behavioural, and genetic risk factors. Additionally, factors such as hospitalization, infections, fractures or injuries, and particular surgical procedures, especially those related to hip and knee replacements or hip fractures, can exacerbate the risk, with incidence rates potentially reaching 60% in these contexts [4]. Furthermore, increased risk of VTE events was observed during the initial week following major surgery or significant trauma [5]. Limb replantation for severe open fractures, such as mangled lower extremity injuries, necessitates extensive and complex reconstructive surgery, often resulting in prolonged hospitalization. In this report, we described two cases of type IIIC open fracture, during which concomitant DVT and PE developed within 72 hours following major reconstruction and limb replantation.

## Case presentation

Case 1

**Figure 1:**
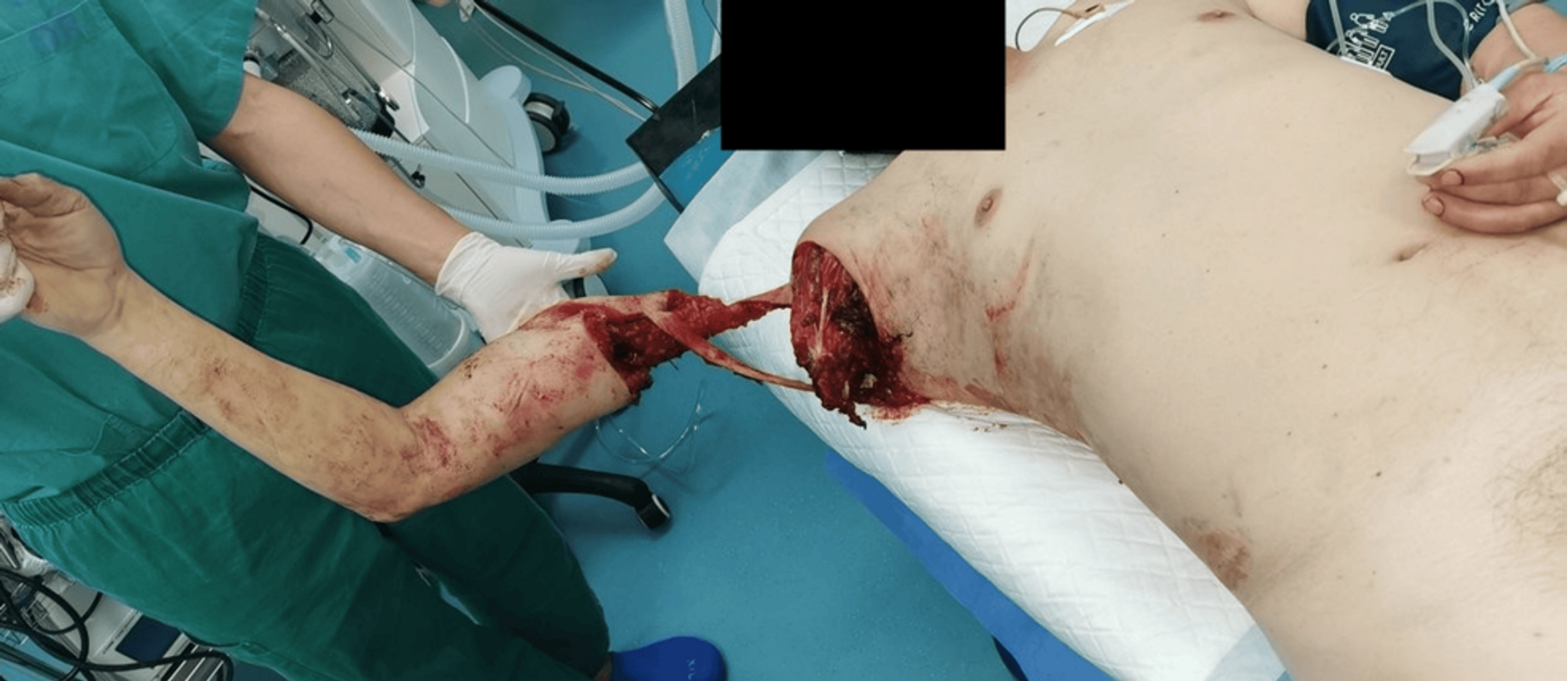
Case 1: Initial presentation of the patient at the time of admission

A 52-year-old healthy man sustained a crush injury while operating machinery. Physical evaluation identified Gustilo-Anderson (GA) type IIIC open fracture at the right glenohumeral joint (Figure 1).

**Figure 2:**
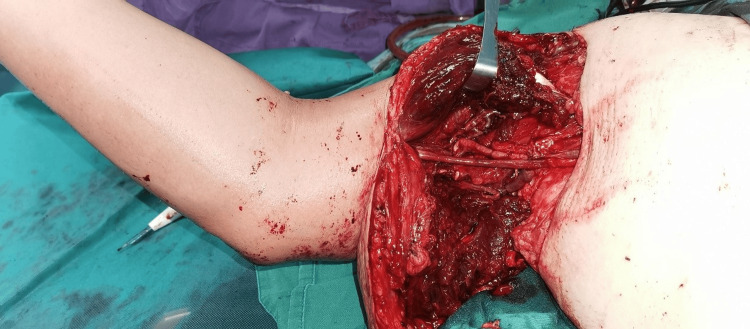
Case 1: The patient underwent surgical debridement, forearm fasciotomies, vascular repair, and limb replantation

The Mangled Extremity Severity Score (MESS) [6] recorded was 9. Chest X-ray and electrocardiogram (ECG) were unremarkable, whereas clotting factors and D-dimer assay were normal. Surgical debridement, forearm fasciotomies, vascular repair, and limb replantation were done (Figure 2).

**Figure 3:**
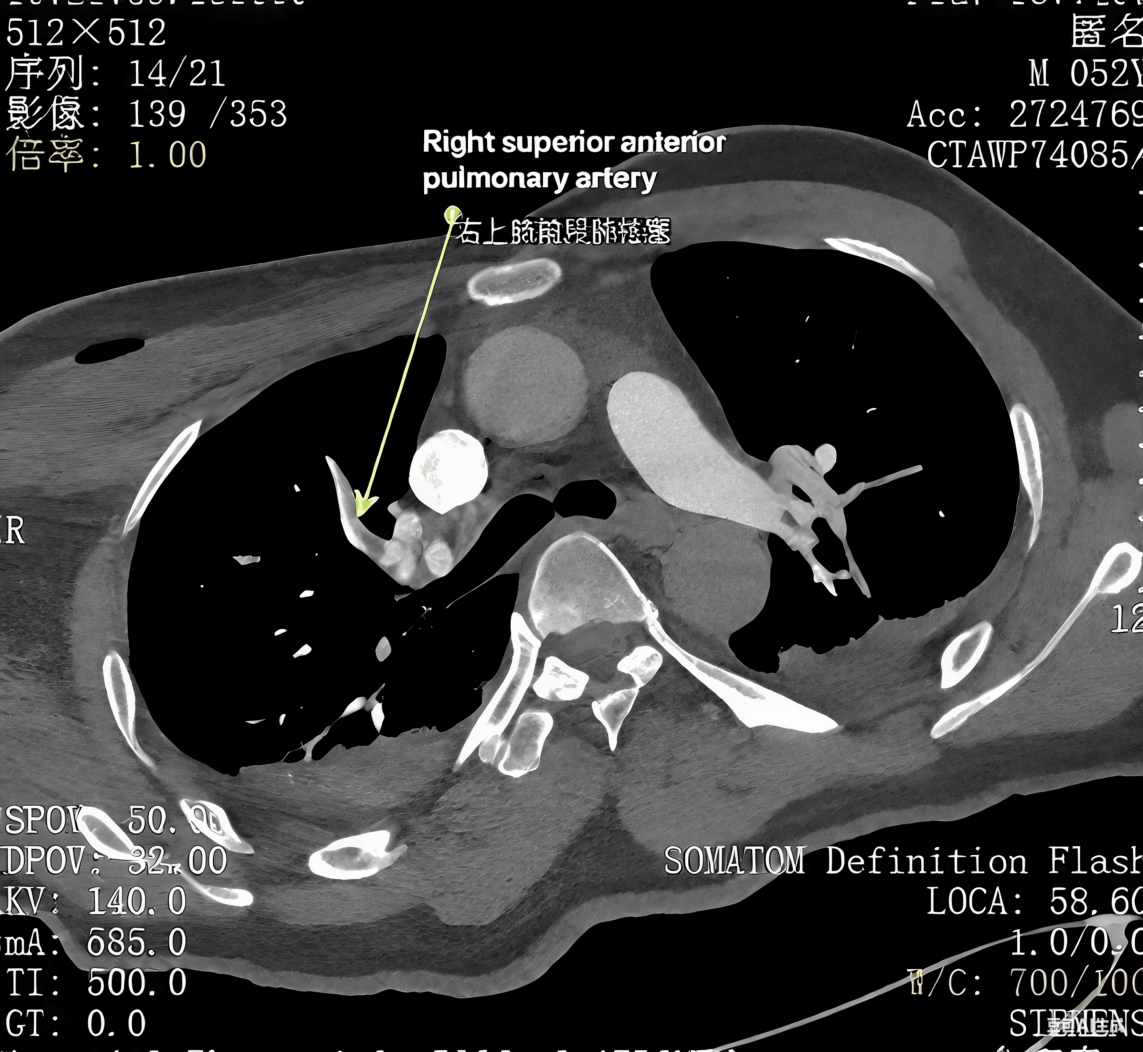
Computed tomography angiography (CTA) of the pulmonary arteries in Case 1, consistent with pulmonary embolism (PE) (arrow)

Negative pressure wound therapy (NPWT) was applied to optimize wound closure. The antibiotic regimen consists of initial IV cefuroxime 250 mg on the day of admission and is changed to levofloxacin 0.5 g based on culture results. Due to the risk of bleeding from NPWT, dextran was administered instead of heparin, despite a Caprini score [7] of 8. On the third postoperative day, PE and DVT were diagnosed following clinical symptoms of dyspnea and delirium, an elevated D-dimer level of 1.37 mg/L FEU, and findings of thrombosis in the pulmonary arteries of the right upper lobe and an axillary vein on computed tomography angiography (CTA) and venography (CTV), respectively (Figure 3).

Antithrombotic therapy of 5000 IU low-molecular-weight heparin (LMWH) was administered daily for a duration of eight days. Following the resolution of VTE, a daily oral administration of 10 mg rivaroxaban was maintained for an additional three months. Subsequently, an anterolateral thigh perforator free flap was used to repair the defect and provide soft tissue coverage. The patient was discharged in satisfactory condition after six weeks.

Case 2

**Figure 4:**
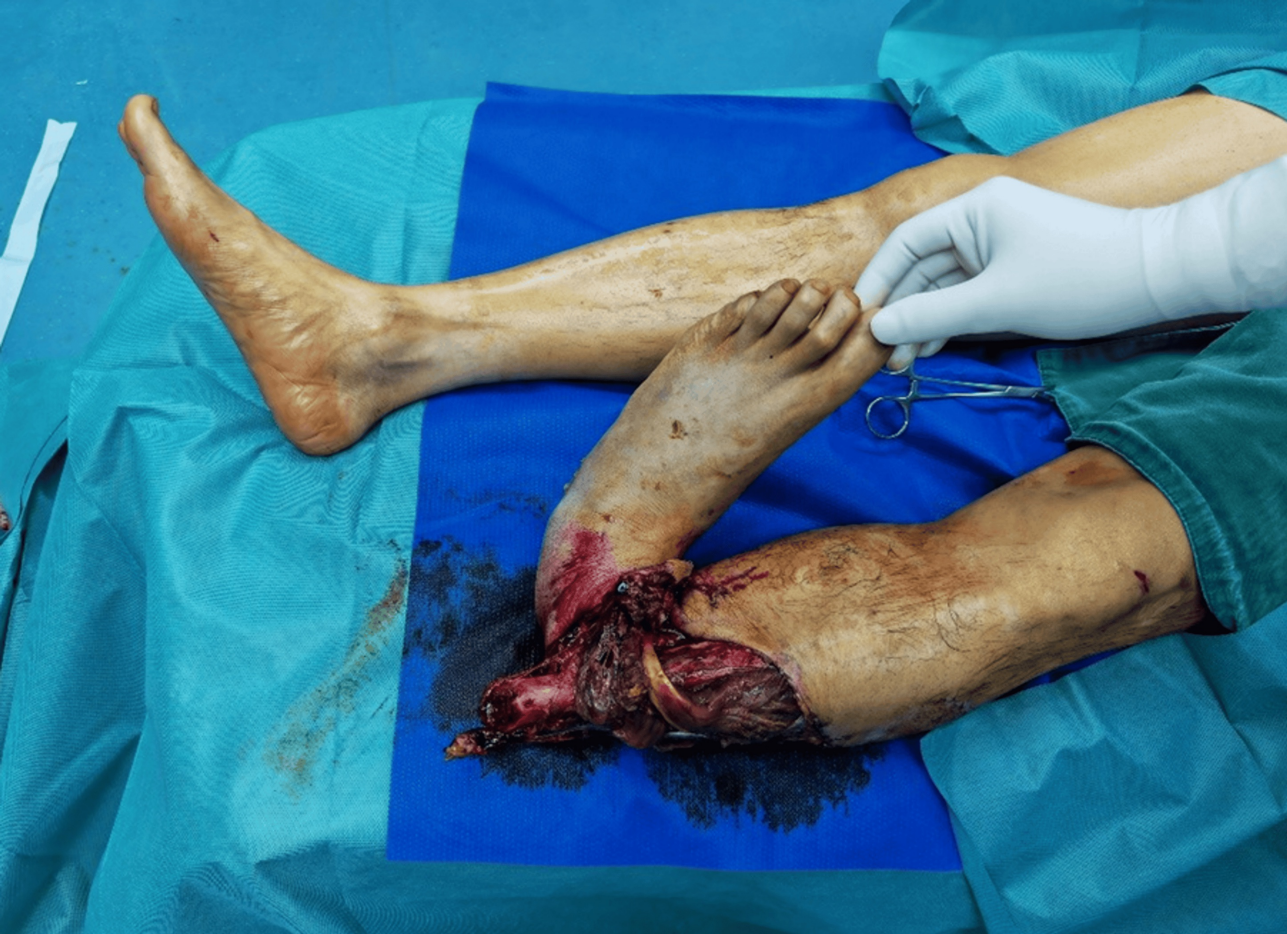
Case 2: Initial presentation of the patient at the time of admission

A 48-year-old healthy male was admitted with a degloving injury resulting from a road traffic accident. Physical examination indicated a GA type IIIC open fracture at the distal tibia, adjacent to the talocrural joint (Figure 4).

**Figure 5:**
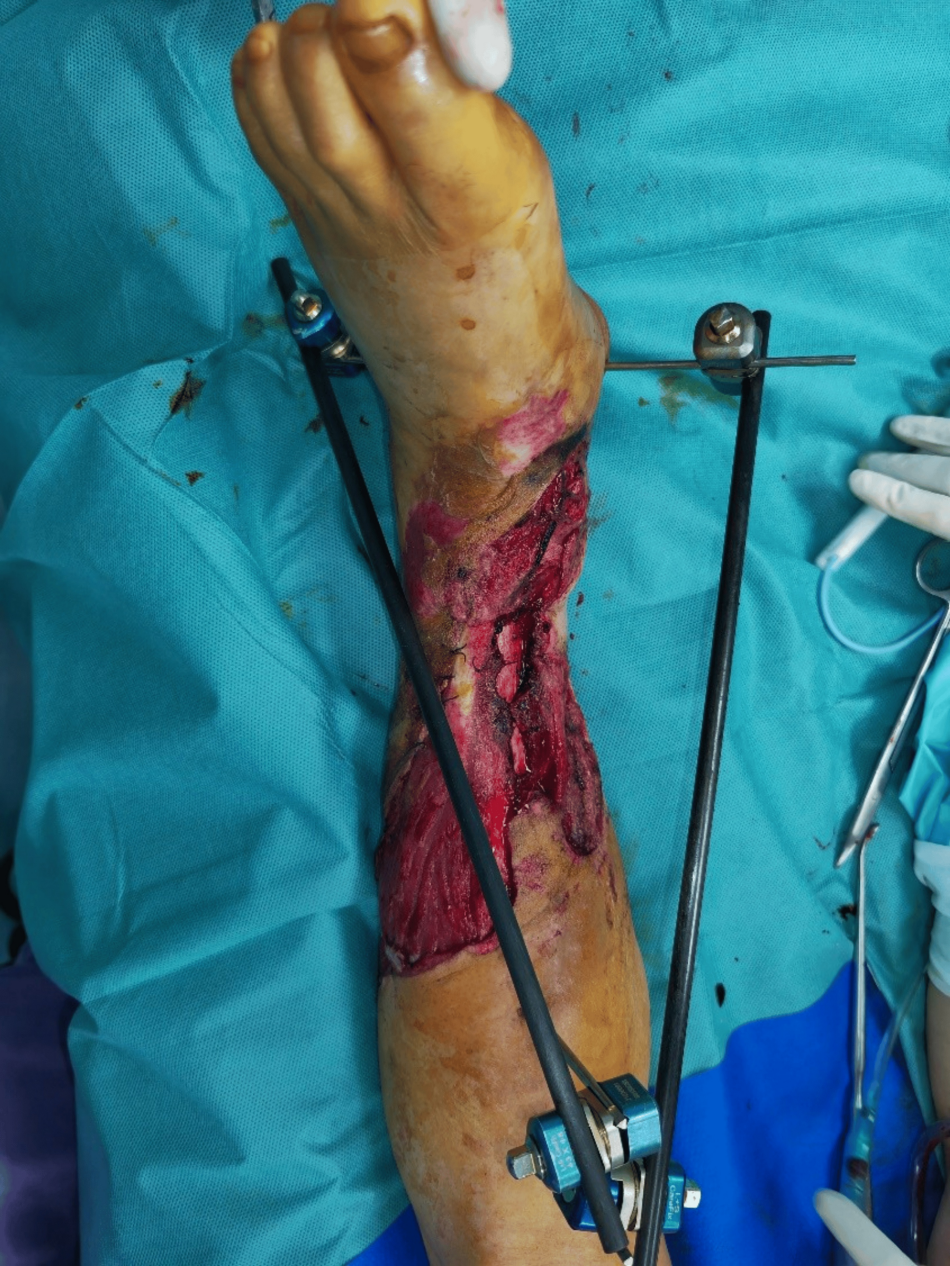
Case 2: The patient underwent surgical debridement, open reduction and internal fixation (ORIF) of the tibiofibular fracture, and stabilization with kickstand external fixation

The MESS score was 8. Chest X-ray and blood tests revealed no notable abnormalities. The patient underwent surgical debridement, open reduction and internal fixation (ORIF) of the tibiofibular fracture, stabilization with kickstand external fixation, and repair of the neurovascular structure (Figure 5).

**Figure 6:**
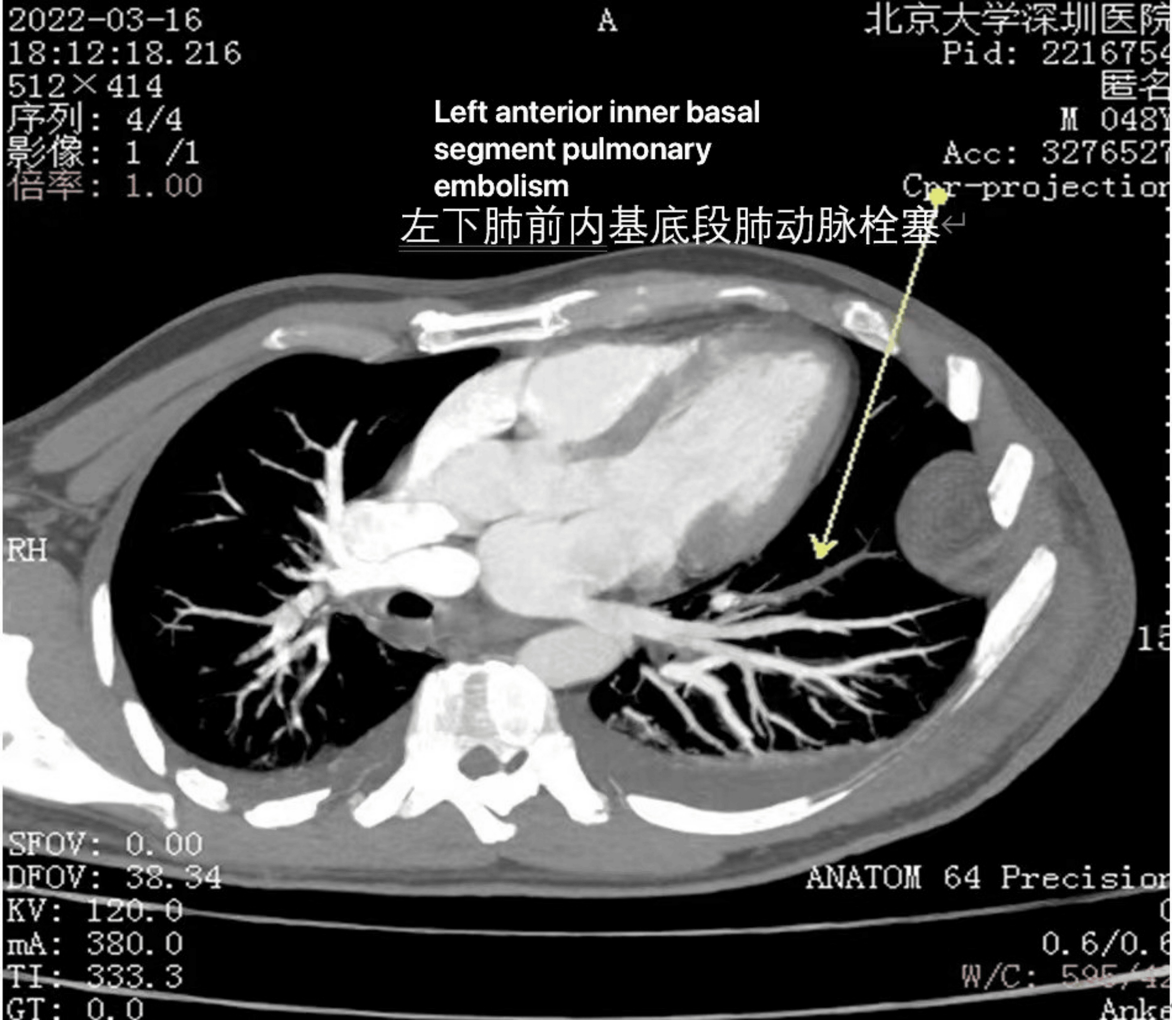
Computed tomography angiography (CTA) of Case 2 showing pulmonary embolism (PE) (arrow)

**Figure 7:**
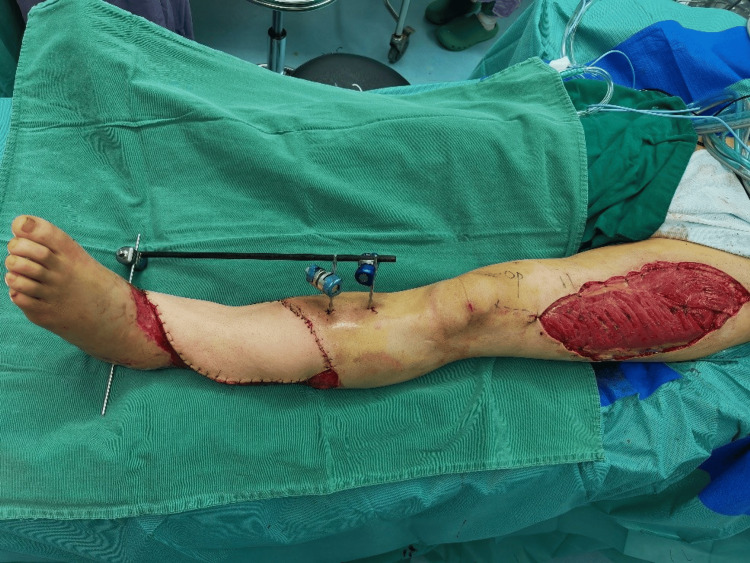
Case 2: The patient underwent anterolateral thigh perforator free flap transplantation for wound coverage of an extensive soft tissue defect

The antibiotic regimen included IV cefuroxime 250 mg at the time of admission, later modified to ceftriaxone 250 mg and levofloxacin 0.5 g based on culture sensitivity results. NPWT was used to prepare the wound bed for the subsequent free flap and skin graft. At 17:16 on the second day, the patient suddenly experienced dyspnea, chest tightness, and profuse sweating. The blood oxygen saturation monitored at the bedside dropped to 94%. Despite a Caprini score of 13, dextran and anisodamine were employed instead of LMWH due to concerns of bleeding while on NPWT. On the second postoperative day, PE and DVT were confirmed on positive findings on CTA (Figure 6) and duplex ultrasonography, clinical symptoms, and an elevated D-dimer assay of 1.12 mg/L FEU. The anticoagulation therapy was adjusted to 5000 IU LMWH over a duration of four weeks, and an inferior vena cava (IVC) filter was placed. VTE events were resolved within three days of prompt intervention. In the second stage, the soft tissue defect was reconstructed utilizing an anterolateral thigh perforator free flap in conjunction with a skin graft from the lower limb (Figure 7).

The patient underwent monitoring for one year, during which the limb exhibited satisfactory condition.

## Discussion

Among hospitalizations in China, the incidence rate of VTE increased by fivefold to 17.5 per 100,000. Deaths associated with PE and DVT were three times more frequent than those associated with DVT alone [8]. Patients of older age, malignancy, ICU stay, and undergoing major surgery require more intensive hospital-acquired venous thromboembolism (HA-VTE) surveillance and prevention [9].

Despite a significant reduction in VTE attributed to heightened awareness and advancements in imaging techniques, clinical symptoms may be overlooked in favour of alternative diagnoses, including atelectasis post-general anaesthesia, pneumonia, or soft tissue infection.

A mangled extremity injury signifies the most severe form of damage to soft tissue and musculoskeletal structures. Amputation is frequently favoured over limb salvage due to the complexity of replantation and the potential for extended rehabilitation periods [10]. VTE and flap thrombosis are among the common perioperative complications associated with major reconstructive and free flap surgeries [11]. While some suggested endothelial wall injury, anastomosis-induced vasospasm, hematoma presence, utilization of vein grafts, and suturing techniques may play a role in its development, questions remain on the causes of thrombosis in microsurgery [12].

Accurate diagnosis of VTE can facilitate timely treatment and enhance patient outcomes. Historically, pulmonary angiography was regarded as the gold standard in diagnosing PE; however, it has now been replaced by pulmonary CTA [13]. In the evaluation of DVT, ultrasonography and CTV with blood investigation of arterial blood gas analysis, clotting factor tests, and D-dimer assays are essential [14]. The extensive soft tissue reconstruction and depth of vascular injury, along with prolonged postoperative immobilization, heightened the risk of VTE events. Upon the patient's development of pulmonary symptoms, including dyspnoea and chest tightness, an immediate pulmonary CTA was conducted, which subsequently revealed findings consistent with PE. We advanced our study by performing CTV on the lower limb and conducting additional blood investigations. DVT was confirmed in CTV, accompanied by elevated D-dimer levels. Although the co-occurrence of PE and DVT is relatively rare, we acknowledged the necessity for a proactive treatment strategy. The patient initiated anticoagulation therapy to address both conditions and mitigate the risk of additional complications.

Upon confirmation of VTE events, the conventional protocol entails the extended-duration heparin infusion, either unfractionated or LMWH, succeeded by the administration of vitamin K antagonists (VKAs) [15]. This approach has been supplanted by short-duration LMWH and followed by direct oral anticoagulants (DOACs), including factor Xa inhibitors like rivaroxaban and thrombin inhibitors such as dabigatran [15,16]. The use of thrombolytics is discouraged due to their association with an elevated risk of bleeding. These changes have resulted in a reduced duration of hospitalization [16]. The anticipated high risk of bleeding resulting from extensive soft tissue defects after surgical debridement and the application of NPWT has led to the administration of the mild anticoagulant dextran. Following the identification of VTE, the antithrombotic regimen was adjusted to LMWH in accordance with clinical guidelines.

In addition to the antithrombotic regimen, we employed an IVC filter to prevent further recurrence of PE and improve the management of VTE events. The placement of the IVC filter was not pursued in the first case due to the thrombus's origin in the axillary veins, which complicated both placement and removal. Moreover, the patient exhibited a positive response to medical management with LWMH. In the second case, the medical team considered placing an IVC filter because the patient was scheduled to undergo subsequent orthopedic procedures that could increase the risk of future embolism. The filter was removed four weeks later following the placement of the perforator free flap.

## Conclusions

In summary, our cases exemplify the difficulties faced in microsurgery. Timely and accurate identification and management are essential. CTA should be performed to exclude the possibility of PE when clinical symptoms arise. Anticoagulation protocols require careful consideration in the context of staged reconstructive procedures.
